# Longitudinal association between the hearing level and quality of life among older adults in south Korea: ascertaining the mediating effect of instrumental activities of daily living using latent growth and autoregressive cross-lagged models

**DOI:** 10.1186/s12889-026-28930-9

**Published:** 2026-08-10

**Authors:** Su Jeong Yang, Jee-Won Kim, Mina Park

**Affiliations:** 1 College of Nursing, Gumi University, 37 Yaeun-ro, Gumi-si, Gyeongsangbuk-do 39213 Republic of Korea; 2 Department of Nursing, Daedong University, Busan, Republic of Korea

**Keywords:** Activities of daily living, Aged, Hearing loss, Longitudinal studies, Quality of life

## Abstract

**Background:**

Hearing loss is increasingly recognized as an important public health concern among older adults and is associated with decreased functional independence and quality of life. However, most previous studies have used cross-sectional designs, limiting understanding of longitudinal relationships and mediating mechanisms. This study examined the longitudinal associations among hearing level, instrumental activities of daily living, and quality of life in older adults and investigated whether instrumental activities of daily living mediated the relationship between hearing level and quality of life.

**Methods:**

This descriptive longitudinal study analyzed data from 3,398 adults aged ≥ 65 years who participated in the 2020, 2022, and 2024 waves of the Korean Longitudinal Study of Aging. Hearing level, instrumental activities of daily living, and quality of life were assessed using self-reported measures. Data were analyzed using SPSS 26.0 and AMOS 26.0, and latent growth modeling and autoregressive cross-lagged modeling were applied.

**Results:**

Hearing level, instrumental activities of daily living, and quality of life declined significantly over time. Latent growth modeling indicated that a higher initial hearing level was associated with higher initial levels of instrumental activities of daily living and quality of life and with a slower decline in instrumental activities of daily living. The indirect effect of the hearing level on quality of life through instrumental activities of daily living was statistically significant (β = 0.018, *p* < .05). Autoregressive cross-lagged modeling demonstrated a significant longitudinal indirect pathway from hearing level at Time 1 to quality of life at Time 3 through instrumental activities of daily living at Time 2.

**Conclusions:**

Hearing level was longitudinally associated with quality of life, and instrumental activities of daily living significantly mediated this longitudinal association among older adults. These findings suggest that integrated interventions combining hearing management with support for instrumental activities of daily living may be beneficial for promoting healthy aging and quality of life among older adults.

**Supplementary Information:**

The online version contains supplementary material available at https://doi.org/10.1186/s12889-026-28930-9.

## Background

With the rapidly aging population worldwide, hearing loss has become an increasingly important chronic health problem among older adults. According to the World Report on Hearing (2021) published by the World Health Organization (WHO), approximately 1.5 billion people worldwide were living with hearing loss in 2019, of whom 430 million had moderate or greater hearing loss requiring rehabilitation [1]. By 2050, approximately 2.5 billion people, representing one in four individuals worldwide, are projected to experience hearing loss, with a substantial proportion of the burden expected to be concentrated among adults aged ≥ 65 years [1]. Similar increasing trends have been observed in the Republic of Korea. A study using National Health Insurance data reported that the number of patients with hearing loss in the Republic of Korea increased by approximately 55.9% between 2010 and 2020, with a particularly marked increase among older adults aged ≥ 60 years [2, 3]. Accordingly, hearing loss among older adults constitutes an important public health issue owing to its increasing prevalence and disease burden in the context of rapid population aging [1, 5].

Age-related hearing loss is a gradual decline in auditory function [4]. Because it typically develops slowly and without pain, it may not be recognized early, delaying diagnosis and rehabilitative intervention [4]. Limited access to auditory rehabilitation and low rates of hearing aid use may further contribute to inadequate management. Consequently, hearing loss can affect not only sensory function but also cognitive, emotional, and social functioning in later life [5, 6]. Communication difficulties and reduced social participation may, in turn, contribute to social withdrawal and cognitive decline [7].

Quality of life (QoL) is an important indicator of health and well-being in older adults. It is a multidimensional concept encompassing physical, psychological, social, and environmental well-being rather than merely the presence or absence of disease [8]. In later life, QoL is closely related to functional independence, social participation, health status, and sensory function [6, 9]. Moderate or greater hearing loss has been significantly associated with poorer social and emotional QoL and may reduce QoL through communication difficulties, restricted social activity, depression, lower self-esteem, and social withdrawal [6, 10]. Consistent with these findings, older adults with hearing loss have reported more severe depressive symptoms and lower QoL than those without hearing loss [10, 11].

Instrumental activities of daily living (IADL), originally conceptualized by Lawton and Brody, are commonly used to assess the capacity of older adults to live independently [12]. Declines in IADL are associated with greater dependence, institutionalization, and mortality risk [13, 14]. Hearing loss may contribute to such functional decline through cognitive, physical, and social pathways. The increased cognitive effort required to process auditory information may reduce the resources available for other functions [15]. In addition, hearing loss has been associated with physical problems, such as impaired balance and an increased risk of falls [15], while communication difficulties and social withdrawal may reduce activity and further compromise functioning [14]. In addition, hearing-related cognitive decline may impair independent functioning in older adults [15]. Previous studies have linked perceived hearing impairment in older adults with poor IADL and functional limitations with poor health-related QoL [16]. Studies involving older adults in the Republic of Korea have likewise associated hearing loss with limitations in physical activity and social participation, which may subsequently contribute to reduced QoL [10, 17]. Collectively, these findings suggest that hearing loss may affect QoL both directly and indirectly through declining IADL. Because age-related sensory decline can reduce functional independence in later life, IADL may be an important mechanism linking hearing level with QoL. This possibility is further supported by evidence associating hearing loss with poor physical and mental health, cognitive impairment, reduced life satisfaction, and hearing-related QoL [10, 14–16]. Collectively, this evidence provides a strong rationale for examining the mediating effect of IADL on the longitudinal relationship between hearing level and QoL.

However, most previous studies have used cross-sectional designs, limiting assessment of the temporal sequence and causal direction among hearing, IADL, and QoL. Studies have also focused largely on the direct association between hearing loss and QoL, providing limited evidence on longitudinal pathways and mediating processes [10, 16]. Although a few longitudinal studies have examined hearing loss in relation to psychosocial outcomes, including QoL [6, 18], the longitudinal mediating effect of IADL remains insufficiently studied. In particular, few studies have applied latent growth modeling (LGM) and autoregressive cross-lagged modeling (ARCLM) to jointly examine longitudinal changes, directional relationships, and IADL-mediated pathways among hearing level, IADL, and QoL in older adults in the Republic of Korea. Because access to hearing rehabilitation and systems of older adult care vary across sociocultural settings, context-specific longitudinal evidence warranted.

Therefore, this study used longitudinal panel data from the Korean Longitudinal Study of Aging (KLoSA) to examine the relationships among hearing level, IADL, and QoL in older adults. Specifically, the study aimed to: (1) describe hearing level, IADL, and QoL among older adults; (2) examine the correlations among these variables; (3) investigate longitudinal changes in hearing level, IADL, and QoL over time; (4) examine the longitudinal effects of hearing level on IADL and QoL; and (5) examine the longitudinal mediating effect of IADL on the relationship between hearing level and QoL.

LGM was used to characterize longitudinal changes in hearing level, IADL, and QoL, whereas ARCLM was applied to investigate the directional relationships and the longitudinal mediating effect of IADL. The findings were intended to inform community-based health management strategies integrating hearing rehabilitation with interventions that support functional maintenance in older adults.

## Methods

### Study design

This descriptive study examined the longitudinal association between hearing level and QoL in older adults and the mediating effect of IADL. The analysis used data from KLoSA collected in 2020, 2022, and 2024. KLoSA, which is conducted by the Korea Employment Information Service (KEIS), is a nationally representative longitudinal panel survey of the sociodemographic characteristics, health, economic activity, and QoL of middle-aged and older adults in the Republic of Korea. Using multistage stratified cluster sampling, the survey recruited community-dwelling adults aged ≥ 45 years nationwide, excluding Jeju Island, and has followed the same participants over time.

### Participants and data collection

Participants were eligible for the present study if they were aged ≥ 65 years in 2020 and completed the 2020, 2022, and 2024 surveys. Trained interviewers collected data using structured questionnaires and computer-assisted personal interviewing (CAPI). Before each interview, participants received an explanation of the study purpose and procedures, and interviews were conducted in a setting intended to facilitate reliable responses.

Of the 4,405 participants aged ≥ 65 years enrolled in the 2020 KLoSA survey, 330 (7.5%) did not participate in 2022 follow-up survey, and an additional 411 (10.1%) did not participate in 2024 follow-up survey, yielding 741 attrition cases (16.8%). Another 266 participants were excluded because of missing data for at least one study variable or covariate. Missing data were handled using listwise deletion (complete-case analysis), resulting in a final analytic sample of 3,398 participants. Baseline characteristics were compared between included participants and those excluded because of attrition or missing data to assess potential selection bias (Supplementary Table 2).

Missing data were handled using complete-case analysis. Participants were included only if they completed the 2020, 2022, and 2024 KLoSA waves and had no missing values for the study variables or covariates. Accordingly, the structural equation models were estimated using a predefined complete-case dataset that included only participants with complete observations across all three survey waves, thereby ensuring a consistent analytic sample across all descriptive and longitudinal analyses. Although full information maximum likelihood (FIML) and multiple imputation are commonly recommended approaches for handling missing data in structural equation modeling (SEM), these methods were not applied because the LGM and ARCLM analyses were conducted using a predefined balanced-panel complete-case dataset, thereby maintaining a consistent analytic sample across all analyses. Complete-case analysis generally provides unbiased parameter estimates under the assumption that data are missing completely at random (MCAR). However, because participants excluded from the analyses differed from those retained with respect to several baseline characteristics, this assumption may not have been fully satisfied. Therefore, the missing-data mechanism may have been more compatible with missing at random (MAR), although the possibility of missing not at random (MNAR) cannot be excluded. Although complete-case analysis reduced the effective sample size, the final analytic sample remained sufficiently large to support the planned longitudinal structural equation modeling analyses. Consequently, the results should be interpreted in light of the potential influence of selection bias and limited external validity.

KLoSA provides sampling weights for its complex survey design; however, weights were not applied because the primary objective was to estimate longitudinal relationships using latent growth and autoregressive cross-lagged models in AMOS. The findings should therefore be interpreted cautiously, because the unweighted parameter estimates may not be fully representative of the target population.

### Measures

#### Hearing level

Hearing level was assessed using a self-reported hearing item in KLoSA. Participants were asked, “How is your hearing?” Responses were originally recorded on a 5-point Likert scale ranging from 1 (“very good”) to 5 (“very poor”). Scores were reverse-coded to ensure that higher values represented better hearing, with 1 indicating “very poor,” 2 “poor,” 3 “fair,” 4 “good,” and 5 “very good.” This standardized item has been used in previous KLoSA-based studies to assess perceived hearing status among older adults [19, 20]. Similar single-item self-reported measures have shown reasonable agreement with objective audiometric assessments, supporting their use in large population-based epidemiological studies [21].

#### IADL

IADL was measured using the Korean Instrumental Activities of Daily Living (K-IADL), developed and validated for Korean older adults by Won et al. [23] based on the Instrumental Activities of Daily Living scale originally developed by Lawton and Brody [12]. The K-IADL assesses 10 domains of instrumental functioning: grooming, housekeeping, meal preparation, laundry, visiting nearby places, transportation use, shopping, financial management, telephone use, and medication management [22]. In KLoSA, each item was coded as 1 (“independent”), 3 (“partially dependent”), or 5 (“completely dependent”), with higher scores indicating greater dependence. For this study, responses were reverse-coded as 1 (“completely dependent”), 2 (“partially dependent”), or 3 (“independent”), to ensure that higher scores indicated greater independence. Scores were summed across the 10 items (range, 10–30). Won et al. reported a Cronbach’s α of 0.94 for the K-IADL [23]; in this study, the Cronbach’s α was 0.95 in 2020, 0.95 in 2022, and 0.96 in 2024. 

#### QoL

QoL was measured using a single KLoSA item: “Compared with others of your age, how satisfied are you with your overall quality of life (happiness)?” Responses were recorded from 0 to 100 in 10-point increments, with higher scores indicating better QoL. For analysis, scores were linearly transformed to a 0–10 scale; this transformation preserved the rank and equal intervals of the original response categories and did not alter interpretation. Although QoL was measured with one item, global single-item measure has been described as a simple and suitable approach for assessing overall QoL [24]. This item has also been used in previous KLoSA-based studies of older adults [25, 26].

#### Demographic characteristics

Demographic characteristics included sex, age, educational level, economic status, and family structure. Sex was categorized as male or female, and age was analyzed as a continuous variable. Educational level was classified as elementary school or below, middle school, high school, or college or above. Economic status comprised total household income in the previous year, analyzed continuously, and housing tenure, categorized as owner-occupied, lump-sum deposit lease, monthly rent, or other. Monthly rent with and without a deposit were combined into a single monthly rent category for the analysis. Family structure comprised the number of generations and household size. The number of generations was categorized as one, two, three, four, or other, and household size was analyzed continuously as the number of cohabiting household members, including the participant.

### Ethical considerations

This secondary analysis used publicly available KLoSA data provided by KEIS. The data were obtained and analyzed in accordance with KLoSA data-use procedures and were fully anonymized. The study was conducted in accordance with the Declaration of Helsinki and approved by the Public Institutional Review Board designated by the Ministry of Health and Welfare (IRB No. P01-202606-01-017).

### Data analysis

Data were analyzed using the Statistical Package for the Social Sciences (SPSS) version 26.0 and Analysis of Moment Structures (AMOS) version 26.0. Descriptive statistics were used to summarize participant characteristics and the study variables, and Pearson’s correlation coefficients were used to examine bivariate associations. LGM and ARCLM were applied to assess the longitudinal relationships among hearing level, IADL, and QoL. LGM estimated the intercept and slope of each variable over time to characterize overall trajectories, whereas ARCLM evaluated directional relationships across measurement occasions after accounting for temporal stability. Thus, the two approaches provided complementary information by characterizing both longitudinal change and temporal directional relationships. Autoregressive and cross-lagged effects were estimated, and the mediating effect of IADL on the association between hearing level and QoL was tested. Model fit was assessed using the χ² statistic, comparative fit index (CFI), Tucker–Lewis index (TLI), root mean square error of approximation (RMSEA), and standardized root mean square residual (SRMR). Because χ² is sensitive to large sample sizes, overall model fit was evaluated based primarily on multiple fit indices rather than on χ² alone. Statistical significance was set at *p* < .05. Indirect effects were tested using bias-corrected bootstrapping with 2,000 resamples and 95% confidence intervals. Because the analysis used observed variables—a single-item hearing level measure, a single-item QoL measure, and the total IADL score—longitudinal measurement invariance was not formally evaluated.

## Results

### Demographic characteristics and descriptive statistics of the study variables

Participant characteristics and descriptive statistics are presented in Supplementary Table 1. The final analytic sample comprised 3,398 participants in 2020, of whom 59.2% were women; the mean age was 69.51 ± 8.75 years. Elementary school education or below was the largest educational category (47.3%), and owner-occupied housing was the most common tenure type (87.5%). The mean household income was 3,402.28 ± 4,039.79, and mean household size was 2.88 ± 1.33.

Mean hearing level scores were 3.35 ± 0.75 in 2020, 3.34 ± 0.76 in 2022, and 3.26 ± 0.75 in 2024, indicating a gradual decline. Mean IADL scores also declined, from 2.94 ± 0.25 in 2020 to 2.90 ± 0.30 in 2022 and 2.84 ± 0.39 in 2024. Similarly, the mean QoL scores decreased over time, from 6.31 ± 1.63 in 2020 to 6.29 ± 1.61 in 2022 and 6.10 ± 1.57 in 2024.

Skewness and kurtosis were examined to assess normality. For hearing level and QoL, skewness ranged from − 0.77 to − 0.42 and kurtosis from 0.26 to 0.80. IADL showed substantial non-normality, with skewness from − 5.17 to − 2.96 and kurtosis from 8.55 to 29.15. Therefore, the asymptotically distribution-free (ADF) estimation method was applied instead of maximum likelihood estimation [27] (Supplementary Table 1). ADF estimation was applied consistently to all structural equation models, including the univariate and multivariate LGMs, ARCLM, and mediation analyses.

Baseline characteristics differed between participants included in the final analysis and those excluded because of attrition or missing data (Supplementary Table 2). Included participants were generally younger and had higher educational attainment, annual household income, household size, hearing level, IADL, and QoL.

### Correlations among hearing level, IADL, and QoL

Correlations among hearing level, IADL, and QoL are presented in Supplementary Table 3. Each variable was positively correlated across the three measurement waves. Hearing level was also positively correlated with IADL and QoL, and IADL was positively correlated with QoL, at each wave (2020, 2022, and 2024).

### Results of the longitudinal mediation analysis using latent growth modeling

Model fit for latent growth modeling was considered acceptable when CFI and TLI were > 0.90, and RMSEA and SRMR < 0.08 [28]. 

#### LGM for hearing level

The LGM for hearing level demonstrated good fit (CFI = 0.996, TLI = 0.989, RMSEA = 0.045, 90% confidence interval [CI] = 0.020–0.077; SRMR = 0.012. All fit indices satisfied the predefined criteria, indicating that the model adequately represented the longitudinal trajectory of hearing level. The initial mean of hearing level was 3.36 and the slope mean was − 0.094, indicating a significant gradual decline in hearing level over the study period. Intercept variance was significant, whereas slope variance and the correlation between the intercept and slope were not significant (Table 1).

**Table 1:** Parameter Estimates for the LGM of Hearing level, IADL, and QoL

Hearing	Parameter	Unstandardized Estimate	Standard Error	Standardized Estimate
	Mean of Growth Factors			
level	Hearing level Intercept	3.364*	0.012	
	Hearing level Slope	-0.094*	0.014	
	Variances and Covariances			
	Hearing level Intercept	0.259*	0.018	1.000
	Hearing level Slope	0.040	0.035	1.000
	Intercept ↔ Slope	-0.014	0.020	-0.138
IADL	Mean of Growth Factors			
	IADL Intercept	2.941*	0.004	
	IADL Slope	-0.091*	0.005	
	Variances and Covariances			
	IADL Intercept	0.037*	0.002	1.000
	IADL Slope	0.019*	0.005	1.000
	Intercept ↔ Slope	0.014*	0.003	0.518
QoL	Mean of Growth Factors			
	QoL Intercept	6.333*	0.027	
	QoL Slope	-0.209*	0.030	
	Variances and Covariances			
	QoL Intercept	1.401*	0.085	1.00
	QoL Slope	0.555*	0.158	1.00
	Intercept ↔ Slope	-0.398*	0.095	-0.451

*LGM* latent growth model, *IADL* Instrumental Activities of Daily Living, *QoL* Quality of Life; * *p* < .05

#### LGM for IADL

The LGM for IADL demonstrated good fit (CFI = 0.997, TLI = 0.990, RMSEA = 0.057, 90% CI = 0.032–0.088; SRMR = 0.013. All fit indices met the predefined criteria, supporting the adequacy of the latent growth model. The initial mean of IADL was 2.94, and the slope was − 0.091, indicating a significant gradual decline in IADL over the study period. Both intercept and slope variances were significant. In addition, the intercept and slope were positively correlated (*r* = .518, *p* < .05), indicating that participants with higher initial IADL tended to decline more slowly over time (Table 1).

#### LGM for QoL

The LGM for QoL demonstrated good overall model fit, with CFI = 0.994, TLI = 0.982, RMSEA = 0.054 (90% CI = 0.029–0.085), and SRMR = 0.014. These indices indicate that the model provided an acceptable representation of the observed data. The initial mean of QoL was 6.33, and the slope was − 0.209, indicating a significant gradual decline in QoL over the study period. Both intercept and slope variances were significant. The intercept and slope were negatively correlated (*r* = − .451, *p* < .05), indicating that participants with higher initial QoL tended to show steeper declines over time (Table 1).

### Multivariate LGM for mediation analysis

**Fig. 1:**
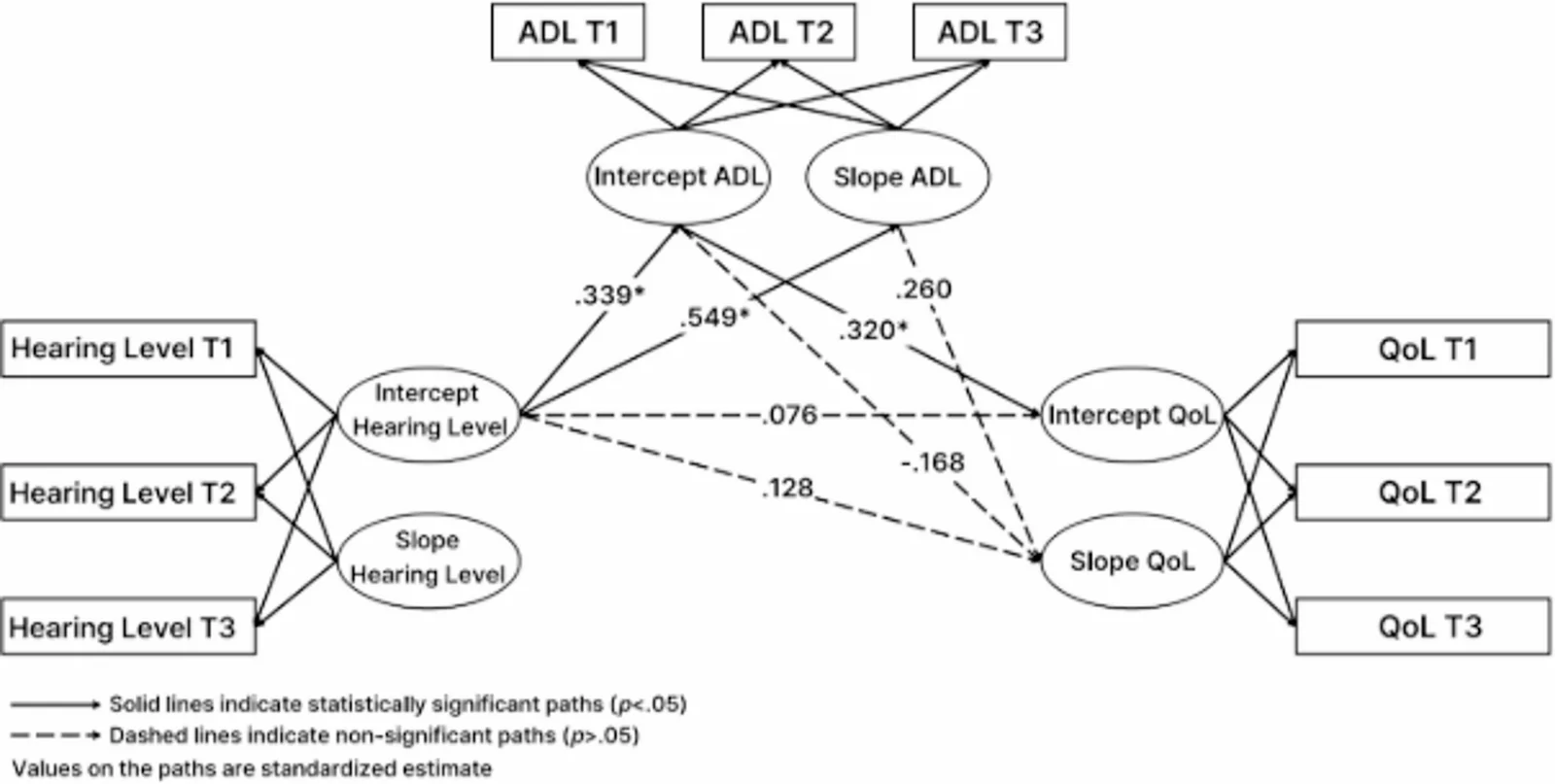
Multivariate Latent Growth Model illustrating the relationships among Hearing level, Instrumental Activities of Daily Living (IADL), and Quality of Life (QoL)

After the univariate growth models were established, a multivariate LGM was used to estimate the relationships among changes in hearing level, IADL, and QoL across the three waves. Model fit was acceptable (CFI = 0.952, TLI = 0.921, RMSEA = 0.028, 90% CI = 0.022–0.035; SRMR = 0.021). A higher hearing level intercept was associated with a higher IADL intercept (standardized coefficient [SC] = 0.339, standard error [SE] = 0.015, *p* < .05) and a higher QoL intercept (SC = 0.076, SE = 0.075, *p* < .05). A higher IADL intercept was also associated with a higher QoL intercept (SC = 0.320, SE = 0.248, *p* < .05). In addition, a higher hearing level intercept was associated with a less negative IADL slope (SC = 0.549, SE = 0.016, *p* < .05), indicating a slower decline in IADL. In contrast, the hearing level intercept was not significantly associated with the QoL slope (SC = 0.128, SE = 0.121, *p* > .05), nor was the IADL intercept (SC = -0.168, SE = 0.353, *p* > .05). The association between the slope of IADL and slope of QoL was also not significant (SC = 0.260, SE = 0.768, *p* > .05) (Table 2; Fig. 1). In addition, the variance of the hearing level slope was estimated as zero; therefore, although the slope factor was retained in the model, structural paths originating from it were not interpreted in the final multivariate model.

**Table 2:** Path Estimates in the Multivariate LGM Linking Hearing level, IADL, and QoL

Path Parameters	Unstandardized Estimate		Standard Error	Standardized Estimate
Hearing level Intercept → IADL Intercept	0.131		0.015	0.339*
Hearing level Intercept → IADL Slope	0.157		0.016	0.549*
IADL Intercept → QoL Intercept	1.959		0.248	0.320*
IADL Intercept → QoL Slope	-0.682		0.353	-0.168
IADL Slope → QoL Slope	1.427		0.768	0.260
Hearing level Intercept → QoL Intercept	0.180		0.075	0.076
Hearing level Intercept → QoL Slope	0.201		0.121	0.128
Path Parameters		Indirect Effect		
Hearing level Intercept → IADL Intercept → QoL Intercept		0.108*		
Hearing level Intercept → IADL Slope → QoL Slope		0.339		

*LGM* latent growth model, *IADL* Instrumental Activities of Daily Living, *QoL* Quality of Life, Indirect effects were estimated using the bootstrap method. *p < .05

Using phantom variables, the mediating effect of IADL on the relationship between hearing level and QoL was examined. The indirect effect of the intercept of hearing level on the intercept of QoL through the intercept of IADL was statistically significant (*β* = 0.108, *p* < .05). Specifically, higher hearing level was associated with higher IADL, which in turn, was associated with higher QoL. However, the indirect effect of the intercept of hearing level on the slope of QoL through the slope of IADL was not statistically significant.

### Results of the longitudinal mediation analysis using ARCLM

To verify the stationarity assumption of the research model, the fit indices of 10 competing models were compared. Model 1 was unconstrained; Models 2–4 imposed equality constraints on autoregressive coefficients, Models 5–7 on cross-lagged coefficients, and Models 8–10 on error covariances. Model selection was based on χ² difference tests and changes in CFI, TLI, and RMSEA because the χ² difference test is sensitive to sample size. CFI and TLI remained > 0.90 and RMSEA remained < 0.08 across the constrained models, supporting stationarity [28]. Model 10, which imposed the most equality constraints and provided the most parsimonious structure with higher degrees of freedom, was therefore selected as the final model for subsequent analyses. The constraints did not substantially worsen model fit while yielding a more parsimonious representation of the longitudinal relationships.

**Fig. 2:**
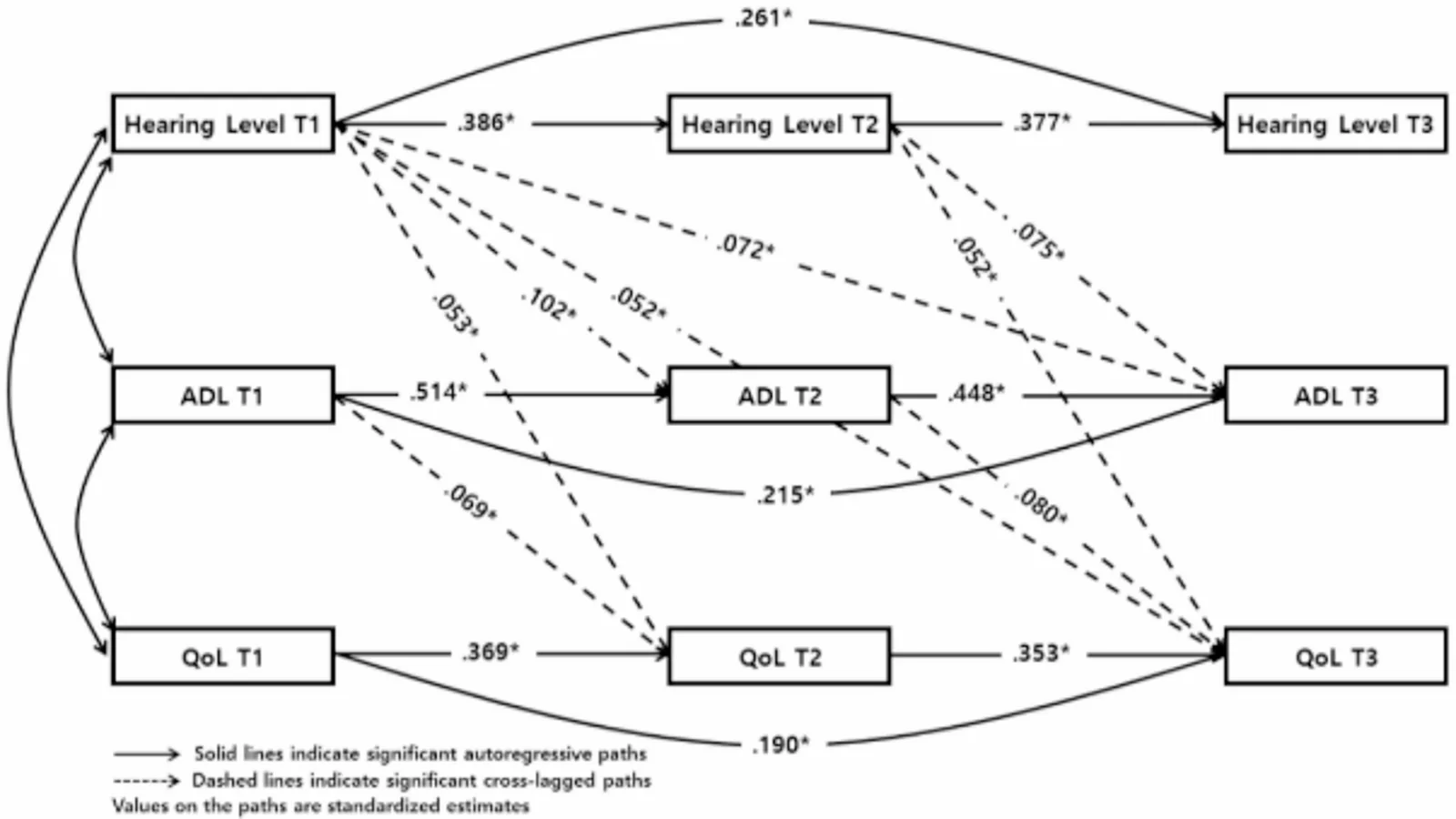
Final Autoregressive Cross-Lagged Model illustrating the autoregressive cross-lagged relationships among hearing level, Instrumental Activities of Daily Living (IADL), and Quality of Life (QoL) across the three measurement waves

The path coefficients of the ARCLM for QoL are presented in Table 3; Fig. 2. Hearing level showed significant autoregressive effects: hearing level at Time 1 predicted hearing level at Time 2 (*β* = 0.386, *p* < .05) and Time 3 (β = 0.261, *p* < .05), and hearing level at Time 2 predicted hearing level at Time 3 (*β* = 0.377, *p* < .05).

**Table 3:** Parameter Estimates for Final ARCLM (Model 10)

Path Parameters	Unstandardized Estimate	Standard Error		Standardized Estimate
Hearing level T1 → Hearing level T2	0.385	0.011		0.386*
Hearing level T1 → Hearing level T3	0.264	0.015		0.261*
Hearing level T1 → IADL T2	0.039	0.005		0.102*
Hearing level T1 → IADL T3	0.037	0.007		0.072*
Hearing level T1 → QoL T2	0.110	0.024		0.053*
Hearing level T1 → QoL T3	0.109	0.033		0.052*
IADL T1 → IADL T2	0.602	0.013		0.514*
IADL T1 → IADL T3	0.339	0.022		0.215*
IADL T1 → QoL T2	0.439	0.065		0.069*
QoL T1 → QoL T2	0.355	0.011		0.369*
QoL T1 → QoL T3	0.184	0.015		0.190*
Hearing level T2 → Hearing level T3	0.385	0.011		0.377*
Hearing level T2 → IADL T3	0.039	0.005		0.075*
Hearing level T2 → QoL T3	0.110	0.024		0.052*
IADL T2 → IADL T3	0.602	0.013		0.448*
IADL T2 → QoL T3	0.439	0.065		0.080*
QoL T2 → QoL T3	0.355	0.011		0.353*
Path Parameters			Indirect Effect	
Hearing level T1 → IADL T2 → QoL T3			0.017*	

*ARCLM* Autoregressive Cross-Lagged Model,* IADL* Instrumental Activities of Daily Living, *QoL* Quality of Life, *p < .05

IADL and QoL also showed significant autoregressive effects. IADL at Time 1 predicted IADL at Time 2 (*β* = 0.514, *p* < .05), and IADL at Time 2 predicted IADL at Time 3 (*β* = 0.448, *p* < .05). QoL at Time 1 predicted QoL at Time 2 (*β* = 0.369, *p* < .05) and Time 3 (β = 0.190, *p* < .05), and QoL at Time 2 predicted QoL at Time 3 (*β* = 0.353, *p* < .05).

Cross-lagged paths from hearing level to subsequent IADL were significant. Specifically, higher hearing level at Time 1 predicted higher IADL at Time 2 (*β* = 0.102, *p* < .05) and Time 3 (*β* = 0.072, *p* < .05), and hearing level at Time 2 predicted IADL at Time 3 (*β* = 0.075, *p* < .05).

Hearing level also predicted subsequent QoL. Hearing level at Time 1 predicted QoL at Time 2 (*β* = 0.053, *p* < .05) and Time 3 (*β* = 0.052, *p* < .05), and hearing level at Time 2 predicted QoL at Time 3 (*β* = 0.052, *p* < .05). Similarly, higher IADL at Time 1 predicted higher QoL at Time 2 (*β* = 0.069, *p* < .05), and IADL at Time 2 predicted QoL at Time 3 (*β* = 0.080, *p* < .05).

Bootstrapping identified a significant indirect effect of hearing level at Time 1 on QoL at Time 3 through IADL at Time 2 (indirect effect = 0.017, *p* < .05). This represented the only significant indirect pathway identified in the ARCLM analysis. Specifically, a higher hearing level at Time 1 predicted greater IADL independence at Time 2, which in turn predicted higher QoL at Time 3 (Table 3).

## Discussion

This study examined the longitudinal associations between hearing level and QoL among older adults and investigated the mediating effect of IADL in this relationship. Overall, a higher hearing level was associated with better daily functioning and higher QoL, and IADL mediated the longitudinal relationship between hearing level and QoL. These findings are consistent with previous studies suggesting that hearing loss may be associated with influence not only sensory functioning but also everyday functioning and psychosocial well-being in older adults [29, 30]. Hearing loss has been associated with communication difficulties and social isolation, which may negatively affect QoL [6, 29, 30], as well as declines in physical functioning and independence in daily activities [31, 32]. Together, these findings suggest that hearing loss may be associated with a series of functional and psychosocial changes that are linked to poorer QoL in later life.

The LGM analysis showed that hearing level, IADL, and QoL declined significantly over time. The decline in hearing level is consistent with age-related hearing loss, in which auditory function gradually deteriorates with age [4]. IADL and QoL also declined, suggesting that reduced independence in instrumental activities and poor QoL in older adults may be longitudinally associated. These findings are consistent with previous studies indicating that QoL in older adults is a multidimensional concept encompassing physical, sensory, psychological, and social functioning [8, 33], and that declines in everyday functioning and sensory functioning are closely associated with lower QoL [16, 33, 34]. The significant intercept variances for hearing level, IADL, and QoL indicated individual differences in their initial levels. In contrast, the variance of the slope for hearing level was not statistically significant, suggesting that although participants differed in their initial hearing levels, their rates of hearing decline were relatively similar. However, the significant slope variances for IADL and QoL indicated individual differences in their rates of change. These findings suggest that even among older adults with similar hearing levels, longitudinal changes in IADL and QoL may vary depending on factors such as physical activity, chronic conditions, and social support, underscoring the potential importance of maintaining physical activity and social engagement in relation to trajectories of everyday functioning and QoL despite age-related hearing decline [5, 13, 35].

The multivariate LGM analysis showed that the intercept of hearing level was positively associated with both the intercepts of IADL and QoL, indicating that the three variables were interrelated at baseline. Specifically, older adults with better hearing tended to have greater independence in daily functioning and higher QoL than those with poorer hearing. These findings are consistent with previous studies suggesting that hearing loss is associated with poorer physical functioning and reduced functional independence, which may subsequently be associated with lower QoL [16, 31]. The significant indirect pathway through IADL further suggests that IADL may represent a potential functional pathway underlying the longitudinal association between hearing level and QoL. In addition, a higher initial hearing level was associated with a slower decline in IADL, suggesting that better hearing was longitudinally associated with a slower decline in functional independence over time. Taken together, the LGM findings suggest that hearing loss may be longitudinally associated with declines in functioning through cognitive, physical, and psychosocial processes—for example, increased listening effort and cognitive load, impaired balance and fear of falling, and reduced social participation—which may, in turn, be associated with declines in IADL and lower QoL over time [15, 36].

In contrast, neither the pathway from the hearing level intercept to the QoL slope nor that from the IADL intercept to the QoL slope was statistically significant. Thus, although initial hearing level and IADL were associated with overall QoL in older adults, they did not sufficiently explain changes in QoL over time. QoL is a multidimensional concept influenced not only by health status but also by psychosocial factors such as economic status, social relationships, depression, and social participation [8, 33]. Therefore, hearing level and IADL alone may not fully explain long-term changes in QoL. QoL may also be influenced by various external factors, including chronic disease, spousal loss, and changes in social roles [5, 8], which may partly account for the weaker longitudinal association between hearing level and QoL over time. In addition, unmeasured clinical factors, such as cognitive impairment, depressive symptoms, hearing-aid use, and visual impairment, may also have influenced the observed associations, highlighting the possibility of residual confounding. Major life events such as retirement, bereavement, caregiving responsibilities, or changes in living arrangements may alter QoL trajectories independently of hearing level or IADL, thereby attenuating the observed longitudinal associations of hearing and daily functioning with QoL slopes. Methodological factors may also have attenuated the observed associations and contributed to the modest effect sizes and nonsignificant QoL slope pathways. Single-item self-reports may capture subjective perceptions and current emotional states as much as objective status, potentially inflating cross-sectional associations while obscuring more subtle longitudinal changes. Complete-case analysis may have produced a healthier and more socioeconomically advantaged sample with less variable trajectories, potentially underestimating the associations. The absence of sampling weights may also have limited the representativeness of the parameter estimates, further contributing to conservative estimates. Taken together, these findings suggest that interventions aimed at improving QoL in older adults may require comprehensive approaches that incorporate not only hearing-related interventions but also psychological, social, and environmental factors.

The ARCLM analysis showed significant autoregressive effects for hearing level, IADL, and QoL, indicating that earlier levels were associated with subsequent levels. This pattern suggests temporal stability in health and functional status rather than abrupt short-term changes. In addition, hearing level at earlier time points was positively associated with subsequent IADL and QoL, whereas IADL at earlier time points was positively associated with subsequent QoL. In particular, the significant indirect pathway from hearing level at Time 1 to QoL at Time 3 through IADL at Time 2 supports a longitudinal mediating effect of IADL. Specifically, better hearing at baseline was associated with greater functional independence at the subsequent assessment, which in turn predicted higher QoL at follow-up. Thus, hearing level may be associated not only with concurrent QoL but also with later functional trajectories and QoL. These findings suggest that IADL may function as a longitudinal mediator of the association between hearing level and QoL over multiple time points, rather than merely reflecting cross-sectional associations [14, 32, 36]. Therefore, strategies aimed at promoting QoL in older adults may benefit from combining hearing management with support for maintaining or enhancing IADL. The longitudinal mediating effect further suggests that hearing level and IADL may be associated with cumulative changes in QoL beyond the short term. Accordingly, IADL may represent a potential functional pathway underlying the longitudinal association between hearing level and long-term QoL trajectories, rather than merely an intermediate outcome. Nevertheless, these findings should be interpreted with appropriate caution. Although the present study identified significant longitudinal associations among hearing level, IADL, and QoL, clinical factors such as depression, cognitive impairment, multimorbidity, hearing-aid use, and visual impairment may also influence both hearing and QoL [5, 8, 33]. Therefore, residual confounding cannot be excluded and may have partially contributed to some of the observed longitudinal associations. Future longitudinal studies incorporating these clinical factors are warranted to further validate and extend the present findings.

Although several cross-lagged coefficients and the indirect effect identified in the ARCLM were modest, they should be interpreted in the context of longitudinal, population-based research involving older adults. Hearing level, IADL, and QoL generally change gradually; therefore, even modest longitudinal associations may accumulate and become clinically meaningful at the population level. The indirect effect of approximately 0.02 suggests that preservation of IADL accounts for only a small proportion of the association between hearing level and QoL and, in realistic clinical practice, would be expected to yield gradual rather than large immediate improvements in QoL. Thus, the present findings should not be interpreted as suggesting that hearing rehabilitation alone would lead to substantial or rapid gains in overall QoL. Instead, better hearing may be associated with greater preservation of independence in IADL, which may, in turn, be associated with a slower decline in functioning and incremental improvements in QoL over time. Because QoL is influenced by multiple physical, psychological, and social factors, hearing-related interventions may provide incremental benefits when implemented as part of a comprehensive approach that also supports everyday functioning and social participation, rather than producing substantial improvements in QoL on their own.

The present findings also extend previous evidence on the longitudinal mechanisms linking hearing level and QoL. Previous studies have primarily focused on the direct relationship between hearing loss and QoL [6, 17, 29]. In contrast, this study identified IADL as a longitudinal mediator of the association between hearing level and QoL. This finding suggests that hearing management may be relevant not only to sensory function but also to the ability to live independently in the community. Accordingly, programs for community-dwelling older adults may benefit from combine routine screening for hearing difficulties and functional limitations with timely access to hearing aids or other assistive devices and rehabilitation that supports IADL. Multidimensional interventions that promote physical activity and social participation and provide emotional and community-based support may further reduce the adverse effects of hearing problems on QoL. In the Korean context, policies that expand coverage for hearing assessment and hearing aids, strengthen community-based gerontological nursing services, and integrate hearing care into long-term care and integrated community care systems may support independent living and promoting healthy aging among older adults. However, because hearing level and IADL did not fully explain changes in QoL, these strategies should also address broader psychosocial and environmental determinants.

This study addressed an important limitation of previous cross-sectional research by longitudinally examining the relationships among hearing level, IADL, and QoL using nationwide KLoSA panel data. The combined application of LGM and ARCLM provided complementary information on overall changes and temporal relationships among the variables. Identifying the mediating effect of IADL also provides evidence relevant to community-based gerontological nursing practice and hearing rehabilitation policies. The use of a large, nationally representative sample, repeated measures over three waves, and two longitudinal modeling approaches enhances the rigor of the analysis and provides additional insight into the temporal relationships among the study variables, although further studies are needed to replicate these results using objective hearing assessments and multidimensional QoL instruments. This design is consistent with previous large-scale longitudinal studies examining hearing and health outcomes in older adults.

Despite these strengths, several limitations of this study should be acknowledged. First, hearing level was measured using a single self-reported item rather than objective audiometric assessments, which may have introduced self-report bias and may reflect general health perceptions or emotional status in addition to hearing level. Second, QoL was also assessed using a single-item measure, which may not have fully captured the multidimensional nature of QoL. Third, participants retained in the final analysis were generally younger and had more favorable socioeconomic and health characteristics than those excluded because of attrition or missing data. Potential selection and survivorship bias therefore cannot be ruled out, and the findings may not be fully generalizable to the broader population of older adults. The longitudinal associations may consequently have been underestimated. Fourth, this study did not include demographic characteristics or several potentially important covariates, such as depressive symptoms, cognitive function, and comorbid chronic conditions in the longitudinal models; therefore, the possibility of residual confounding cannot be excluded. These factors may have partly explained the observed longitudinal associations among hearing level, IADL, and QoL. Although the hearing item reflected perceived hearing while using hearing aids when applicable, detailed information on the type and duration of hearing aid use, cochlear implants, rehabilitation services, and treatment history was not available in the KLoSA dataset. These factors may also be associated with functional independence and QoL and therefore may have affected the observed associations between hearing level, IADL, and QoL. In addition, the 2020–2024 follow-up period may have been too short to capture longer-term changes in hearing level, IADL, and QoL. Furthermore, because part of the study period overlapped with the coronavirus disease 2019 (COVID-19) pandemic (2020–2022), changes in healthcare utilization, daily activities, and social participation during this period may have influenced the observed longitudinal associations. Finally, complete-case analysis and the absence of sampling weights may have further limited population representativeness and contributed to selection bias. Future longitudinal and intervention studies should consider incorporating objective hearing assessments, multidimensional QoL instruments, broader psychosocial and clinical variables, and detailed hearing rehabilitation information. Despite these limitations, this study provides longitudinal evidence on the associations among hearing level, IADL, and QoL and the mediating effect of IADL in older adults in the Republic of Korea. The findings may inform the development of community-based hearing management strategies that support functional independence and promoting healthy aging.

## Conclusion

Using KLoSA data, this study examined the longitudinal associations among hearing level, IADL, and QoL in older adults and the mediating effect of IADL. Hearing level, IADL, and QoL declined significantly over time, and hearing level was longitudinally associated with subsequent IADL and QoL. In addition, IADL mediated the longitudinal association between hearing level and QoL, indicating that hearing level may be related to QoL both directly and indirectly through functional independence among older adults. These findings suggest that hearing loss may be associated with reduced functional independence and poorer overall QoL in older adults, extending beyond sensory impairment alone. Furthermore, the findings suggest that psychosocial and environmental factors, including social participation and emotional status, may contribute to changes in QoL beyond the associations of hearing level and IADL.

Accordingly, supporting QoL in older adults may require integrated strategies that combine hearing management with efforts to maintain IADL and address relevant psychosocial factors. Future studies should evaluate whether integrated interventions combining hearing management with support for IADL are associated with improvements in functional independence and QoL. They should also incorporate objective hearing assessments, multidimensional QoL instruments, and longitudinal analyses of depression, social participation, cognitive function, while adjusting for relevant demographic characteristics. These findings may inform the development of clinical guidelines and health policies for older adults in the Republic of Korea.

## Supplementary Information

Supplementary Material 1: Supplementary table 1. Demographic Characteristics of the Participants and Descriptive Statistics of the Study VariablesSupplementary table 2. Correlations Among Hearing Levels, Activities of Daily Living, and Quality of Life (2020–2024)Supplementary table 3. Comparison of baseline characteristics between participants included in the final analysis and those excluded because of attrition or missing data.

## Data Availability

The datasets analyzed during the current study are publicly available from the Korean Longitudinal Study of Aging (KLoSA) repository operated by the Korea Employment Information Service (KEIS) and are available upon reasonable request and approval from the data provider.

## Abbreviations

IADL: Instrumental Activities of Daily Living
AMOS: Analysis of Moment Structures
ARCLM: Autoregressive Cross-Lagged Modeling
CAPI: Computer-Assisted Personal Interviewing
CFI: Comparative Fit Index
FIML: Full Information Maximum Likelihood
IRB: Institutional Review Board
KEIS: Korea Employment Information Service
KLoSA: Korean Longitudinal Study of Aging
LGM: Latent Growth Modeling
MAR: Missing at Random
MCAR: Missing Completely at Random
MNAR: Missing Not at Random
QoL: Quality of Life
RMSEA: Root Mean Square Error of Approximation
SE: Standard Error
SEM: Structural Equation Modeling
SPSS: Statistical Package for the Social Sciences
SRMR: Standardized Root Mean Square Residual
TLI: Tucker–Lewis Index
WHO: World Health Organization
