# Supplementary material for: Longitudinal association between the hearing level and quality of life among older adults in south Korea: ascertaining the mediating effect of instrumental activities of daily living using latent growth and autoregressive cross-lagged models
Source: BMC Public Health. 2026 Aug 10;26:3225. doi: 10.1186/s12889-026-28930-9 (PMC13741695; doi:10.1186/s12889-026-28930-9)
Supplement: Supplementary file 1 [file 12889_2026_28930_MOESM1_ESM.docx]

Supplementary 1. Demographic Characteristics of the Participants and Descriptive Statistics of the Study Variables

(N=3,398)

| Variables | | | Categories | | N (%) or Mean ± SD | |
| --- | --- | --- | --- | --- | --- | --- |
| Sex | | | Male | | 1388 (40.8) | |
|  | | | Female | | 2010 (59.2) | |
| Age (years) | | |  | | 69.51 ± 8.75 | |
| Educational level | | | Elementary school or below | | 1607 (47.3) | |
|  | | | Middle school | | 637 (18.7) | |
|  | | | High school | | 843 (24.8) | |
|  | | | College or above | | 311 (9.2) | |
| Annual household income  (10,000 KRW) | | |  | | 3402.28 ± 4039.79 | |
| Housing tenure | | | Owner-occupied | | 2974 (87.5) | |
|  | | | lump-sum deposit lease | | 173 (5.1) | |
|  | | | Monthly rent | | 230 (6.8) | |
|  | | | Others | | 21 (0.6) | |
| Household size (persons) | | |  | | 2.88 ± 1.33 | |
| Variables | Year | Variable Name | | Mean±SD | Skewness | Kurtosis |
| Hearing | 2020 | Hearing level T1 | | 3.35±0.75 | -0.52 | 0.58 |
| level | 2022 | Hearing level T2 | | 3.34±0.76 | -0.42 | 0.33 |
|  | 2024 | Hearing level T3 | | 3.26±0.75 | -0.58 | 0.26 |
| IADL | 2020 | IADL T1 | | 2.94±0.25 | -5.17 | 29.15 |
|  | 2022 | IADL T2 | | 2.90±0.30 | -4.13 | 18.38 |
|  | 2024 | IADL T3 | | 2.84±0.39 | -2.96 | 8.55 |
| QoL | 2020 | QoL T1 | | 6.31±1.63 | -0.77 | 0.62 |
|  | 2022 | QoL T2 | | 6.29±1.61 | -0.77 | 0.80 |
|  | 2024 | QoL T3 | | 6.10±1.57 | -0.68 | 0.73 |

Note. SD = Standard Deviation; IADL = Instrumental Activities of Daily Living; QoL = Quality of Life;T1 = Time 1; T2 = Time 2; T3 = Time 3

Supplementary 2. Comparison of baseline characteristics between participants included in the final analysis and those excluded because of attrition or missing data.

| Characteristic | Included  (n = 3,398) | Excluded  (n = 1,007) | Test statistic | *p* |
| --- | --- | --- | --- | --- |
| Age (years) | 74.55 ± 6.60 | 80.86 ± 7.93 | *t* =22.97 | <.001 |
| Sex |  |  | χ² = 4.96 | .026 |
| Male | 1,388 (40.8) | 451 (44.8) |  |  |
| Female | 2,010 (59.2) | 556 (55.2) |  |  |
| Education level |  |  | χ² =50.10 | <.001 |
| Elementary school or below | 1,607 (47.3) | 603 (59.9) |  |  |
| Middle school | 637 (18.7) | 133 (13.2) |  |  |
| High school | 843 (24.8) | 198 (19.7) |  |  |
| College or above | 311 (9.2) | 73 (7.2) |  |  |
| Annual household income  (10,000 KRW) | 2507.79 ± 3128.91 | 2065.78 ± 2429.88 | *t* = −4.13 | <.001 |
| Housing tenure |  |  | χ² = 18.31 | <.001 |
| Owner-occupied | 2,974 (87.5) | 831 (82.5) |  |  |
| lump-sum deposit lease | 173 (5.1) | 67(6.7) \| |  |  |
| Monthly rent | 230 (6.8) | 96 (9.5) |  |  |
| Other | 21 (0.6) | 13 (1.3) |  |  |
| Household size | 2.63 ± 1.33 | 2.44 ± 1.37 | *t* = −3.96 | <.001 |
| Hearing level | 3.35 ± 0.75 | 3.04 ± 0.80 | *t* = −10.95 | **<.001** |
| IADL | 29.38 ± 2.46 | 26.74 ± 5.86 | *t* = −13.98 | <.001 |
| QoL | 6.31 ± 1.63 | 5.78 ± 1.86 | *t* = −8.16 | **<.001** |

Note. Data are presented as mean ± SD or n (%). *P* values were calculated using independent-samples *t* tests for continuous variables and Pearson's χ² tests for categorical variables.

IADL = Instrumental Activities of Daily Living; QoL = Quality of Life

Supplementary 3. Correlations Among Hearing Level, Activities of Daily Living, and Quality of Life (2020–2024)

| No | Variables | 1 | 2 | 3 | 4 | 5 | 6 | 7 | 8 | 9 |
| --- | --- | --- | --- | --- | --- | --- | --- | --- | --- | --- |
| 1 | Hearing level T1 | 1 |  |  |  |  |  |  |  |  |
| 2 | Hearing level T2 | .442** | 1 |  |  |  |  |  |  |  |
| 3 | Hearing level T3 | .436** | .451** | 1 |  |  |  |  |  |  |
| 4 | IADL T1 | .197** | .186** | .195** | 1 |  |  |  |  |  |
| 5 | IADL T2 | .203** | .273** | .258** | .598** | 1 |  |  |  |  |
| 6 | IADL T3 | .242** | .270** | .347** | .534** | .581** | 1 |  |  |  |
| 7 | QoL T1 | .109** | .072** | .099** | .193** | .179** | .189** | 1 |  |  |
| 8 | QoL T2 | .107** | .138** | .129** | .184** | .227** | .197** | .458** | 1 |  |
| 9 | QoL T3 | .153** | .160** | .217** | .207** | .217** | .284** | .393** | .429** | 1 |

Note. IADL = Instrumental Activities of Daily Living; QoL = Quality of Life; T1 = Time 1; T2 = Time 2; T3 = Time 3; **p*<.05, ** *p*<.01
